# NRF2 Activation Confers Resistance to eIF4A Inhibitors in Cancer Therapy

**DOI:** 10.3390/cancers13040639

**Published:** 2021-02-05

**Authors:** Viraj R. Sanghvi, Prathibha Mohan, Kamini Singh, Linlin Cao, Marjan Berishaj, Andrew L. Wolfe, Jonathan H. Schatz, Nathalie Lailler, Elisa de Stanchina, Agnes Viale, Hans-Guido Wendel

**Affiliations:** 1 Cancer Biology and Genetics Program, Memorial Sloan Kettering Cancer Center, New York, NY 10065, USA; mohanp@mskcc.org (P.M.); singhk2@mskcc.org (K.S.); linlin.cao@epfl.ch (L.C.); berishajm@mskcc.org (M.B.); andrew.wolfe@ucsf.edu (A.L.W.); jschatz@med.miami.edu (J.H.S.); wendelh@mskcc.org (H.-G.W.); 2 Department of Molecular and Cellular Pharmacology, Sylvester Comprehensive Cancer Center, Miller School of Medicine, University of Miami, Miami, FL 33136, USA; 3 Swiss Institute of Experimental Cancer Research, EPFL, 1015 Lausanne, Switzerland; 4 Hellen Diller Comprehensive Cancer Center, University of California, San Francisco, CA 94143, USA; 5 Department of Medicine, Sylvester Comprehensive Cancer Center, Miller School of Medicine, University of Miami, Miami, FL 33136, USA; 6 Integrated Genomics Operation, Marie-Josée and Henry R. Kravis Center for Molecular Oncology, Memorial Sloan Kettering Cancer Center, New York, NY 10065, USA; contact@nathalielailler.com (N.L.); vialea@mskcc.org (A.V.); 7 Department of Antitumor Assessment Core and Molecular Pharmacology, Memorial Sloan Kettering Cancer Center, New York, NY 10065, USA; destance@mskcc.org

**Keywords:** NRF2, KEAP1, drug resistance, eIF4A, silvestrol, G-quadruplex, lymphoma

## Abstract

**Simple Summary:**

eIF4A-targeted translational inhibitors, such as silvestrol and its analogues, have emerged as strong anticancer therapies. Here, we tested the efficacy of eIF4A inhibition across a large and diverse panel of cancer cell lines and found B cell lymphomas to be the most sensitive group. Moreover, we performed a genetic screen and identified NRF2 activation as a major mechanism of resistance to silvestrol and related eIF4A inhibitors. Mechanistically, NRF2 activation broadly increases protein synthesis, and this effect is more pronounced on specific mRNAs that require eIF4A for translation. Finally, blocking NRF2 function by preventing its deglycation restores silvestrol sensitivity in cells that harbor NRF2 activation. Overall, our findings indicate that eIF4A inhibitors are a feasible therapeutic option against lymphoma and other cancers and that NRF2 activation status may be an important predictor of their efficacy.

**Abstract:**

Inhibition of the eIF4A RNA helicase with silvestrol and related compounds is emerging as a powerful anti-cancer strategy. We find that a synthetic silvestrol analogue (CR-1-31 B) has nanomolar activity across many cancer cell lines. It is especially active against aggressive MYC^+^/BCL2^+^ B cell lymphomas and this likely reflects the eIF4A-dependent translation of both MYC and BCL2. We performed a genome-wide CRISPR/Cas9 screen and identified mechanisms of resistance to this new class of therapeutics. We identify three negative NRF2 regulators (KEAP1, CUL3, CAND1) whose inactivation is sufficient to cause CR1-31-B resistance. NRF2 is known to alter the oxidation state of translation factors and cause a broad increase in protein production. We find that NRF2 activation particularly increases the translation of some eIF4A-dependent mRNAs and restores MYC and BCL2 production. We know that NRF2 functions depend on removal of sugar adducts by the frutosamine-3-kinase (FN3K). Accordingly, loss of FN3K results in NRF2 hyper-glycation and inactivation and resensitizes cancer cells to eIF4A inhibition. Together, our findings implicate NRF2 in the translation of eIF4A-dependent mRNAs and point to FN3K inhibition as a new strategy to block NRF2 functions in cancer.

## 1. Introduction

The oncogenic transcription factor NRF2 (Nuclear factor erythroid 2-related factor 2, encoded by the *NFE2L2* gene) drives a cytoprotective redox program centered on glutathione synthesis and reduction [1,2,3,4]. NRF2 is mutationally activated in 10–30% of different solid tumors including liver, lung, stomach, colon and other cancers and mutations typically disrupt the interaction between NRF2 and its E3 ligase KEAP1 (Kelch Like ECH Associated Protein 1) directly or by impeding KEAP1 co-factors CUL3 (Cullin 3) and CAND1 (Cullin Associated and Neddylation Dissociated 1) [2,5,6,7,8]. NRF2 has been implicated in cancer therapeutic responses and neutralizes reactive oxygen species (ROS) that arise during chemo- and radiation treatment [2,9,10,11,12]. Intriguingly, NRF2 also impairs EGFR inhibition, suggesting additional effects beyond ROS neutralization [13,14]. More recently, NRF2 has been implicated in translational and metabolic reprogramming of cancer cells. For example, NRF2 steers glucose to anabolic cancer metabolism and prevents oxidation of multiple translational factors to augment protein output [15,16]. There is great interest in targeting NRF2 function in cancer and we have no direct NRF2 inhibitors. We recently showed that NRF2 stability and function depend on the removal of sugars from the NRF2 protein. The process is called de-glycation and it is triggered when the fructosamine-3-kinase (FN3K) phosphorylates sugar residues that are attached to the NRF2’s C-terminal domain [14].

Cancer cells broadly increase their protein production, and this provides new opportunities for cancer detection, monitoring, and treatment [17,18,19]. The natural product, silvestrol, and related compounds are selective and potent inhibitors of the RNA helicase and translation initiation factor eIF4A (Eukaryotic Translational Initiation Factor 4A, also known as DDX2) [20]. The eIF4A helicase is uniquely required for the translation of mRNAs with long and highly structured 5′ UTRs, including those with guanine tetrad sequences that are thought to fold into G-quadruplex (GQ) structures [21]. Manual polysome fractionation experiments identified some eIF4A-dependent mRNAs such as *MYC*, *BCL2*, and *MCL1* [22,23]. Polysome experiments suffer from variability and sometimes fail to identify significant changes in technical replicates [24]. The newer methods based on ribosome profiling and RNA sequencing provide more accurate and statistically sound data [25]. Specifically, they reveal that less than 1% of the transcriptome depends on eIF4A and, importantly, these eIF4A-dependent genes include key growth and cell death factors such as MYC, BCL2, MCL1, CDK4, CCND3, EZH2, NOTCH1, and others [21,23]. EIF4A inhibitors block this pro-oncogenic translation program and produce powerful anti-cancer effects with limited effects in non-proliferative tissues [21,23]. These properties have led to great interest in this new class of drugs. Here, we examine the effect of a synthetic eIF4A inhibitor (CR-1-31 B) against aggressive, MYC and BCL2 positive lymphomas and we specifically explore potential mechanisms of resistance to the eIF4A inhibitor treatment.

## 2. Results

### 2.1. CR-1-31 B Kills Aggressive Lymphoma Cells at Nanomolar Concentrations

**Figure 1:**
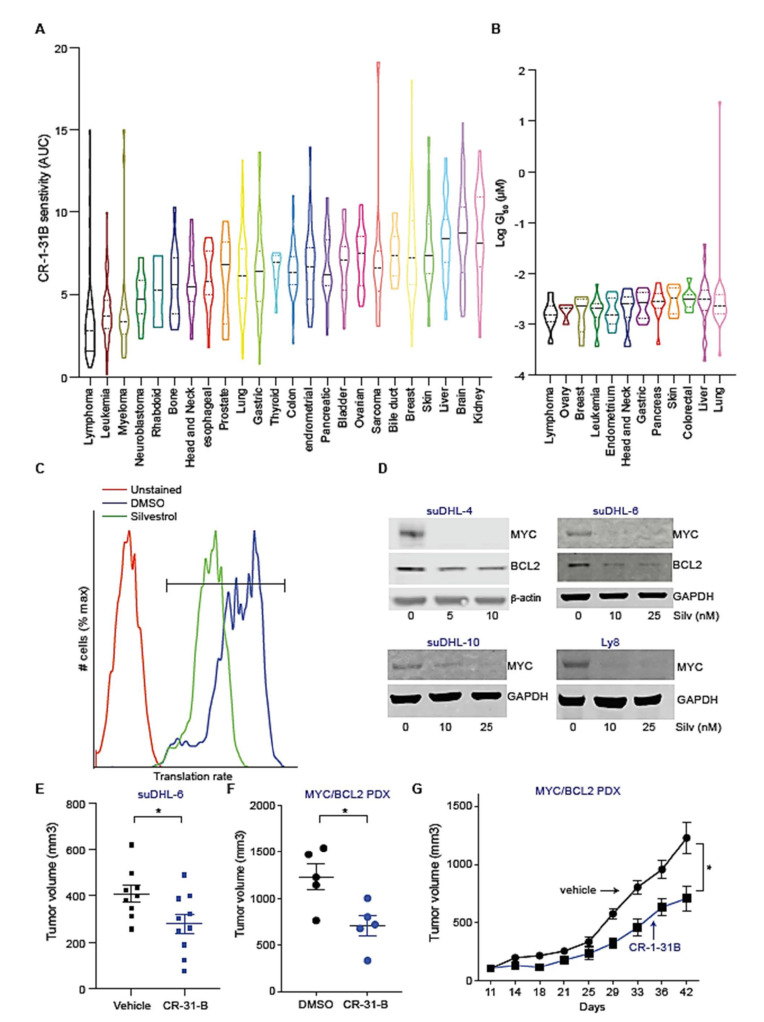
Single agent activity of a synthetic eIF4A inhibitor across multiple tumor types. (**A**) CR-1-31 B sensitivity profile across 770 cell lines generated from the DepMap database; the data are represented as the means of area under the drug response curve (AUC) for indicated cancer types; (**B**) violin plot depicting CR-1-31 B concentration, which leads to 50% growth inhibition (GI_50_) in 183 different cancer lines; (**C**) flow cytometry to measure AHA incorporation in DMSO or silvestrol treated (10 nM, 1 h) suDHL-6 cells; (**D**) immunoblot analysis to measure MYC and BCL2 expression from DMSO or silvestrol treated (24 h) human lymphoma cells, as indicated. Uncropped immunoblot images present in Figure S5; (**E**) tumor volumes of subcutaneously engrafted MYC^+^/BCL2^+^ suDHL-6 after treatment with vehicle or CR-1-31 B; data are shown as mean and standard deviation (s.d.) of tumor volumes ~28 days post implantation from *n* = 10 mice; (**F**,**G**) tumor volumes at day 42 (**F**) and growth kinetics (**G**) of MYC^+^/BCL2^+^ patient-derived DLBCL cells engrafted in NSG (NOD scid gamma) mice and treated with either vehicle or CR-1-31 B, as indicated; means of tumor volumes are plotted and error bars represent s.d. from *n* = 5 mice. * represents *p*-value < 0.05.

Dependency Map (DepMap) is a publicly available repository that has systematically cataloged in vitro sensitivities for several drugs across ~800 cell lines spanning all major tissues [26]. CR-1-31 B, a synthetic silvestrol analog [27], shows very potent single agent activity against many cancer cell lines in vitro with B cell lymphoma lines among the most sensitive with an area under the CR-1-31 B response curve ranging from 0.5737 (Pfeiffer) to 15 (REC1) (Figure 1A). In parallel, we screened 183 cancer cell lines for sensitivity to CR-1-31 B and we also found lymphoma cells to be the most sensitive (Figure 1B). We further confirmed this striking sensitivity across a panel of aggressive diffused large B cell lymphoma (DLBCL) lines and found 50% inhibitory concentration (IC_50_) values in the range of <1 nM (suDHL-4) to 5 nM (SuDHL-10, Ly8, and TMD8) irrespective of their pathological sub-classification (Figure S1A). Interestingly, CR-1-31 B also shows strong activity against lymphoma cells with known MYC and/or BCL2 alterations that continue to represent a therapeutic challenge. Notably, the translation of both MYC and BCL2 depends on eIF4A making these genes targets of the inhibitor [21]. Consistent with a role for translational activation in these aggressive lymphomas, we observe in a small cohort (*n* = 34) of MYC^+^/BCL2^+^ a clear correlation between MYC and BCL2 status and phosphorylation of ribosomal protein kinase p70S6K, an indicator of translation activation (Figure S1B,C). These findings suggest a role for eIF4A inhibitors in the treatment of aggressive lymphomas including the hard to treat MYC^+^/BCL2^+^ disease.

Next, we directly examined how eIF4A inhibition affects translation in MYC^+^/BCL2^+^ lymphomas. Briefly, incorporation of the methionine analog azidohomoalanine (AHA) into peptides reflects global translation levels. Silvestrol treatment (10 nM, ~1 h) causes over 50% decline in AHA incorporation and global protein synthesis in suDHL-6 cells (Figure 1C). Consistent with our previous findings [21], we see that silvestrol blocks the translation of the MYC and BCL2 mRNAs, without affecting the *GAPDH* housekeeping gene, across several lymphoma lines (SuDHL-4, SuDHL-6, SuDHL-10, Ly8) (Figure 1D). This is independent of proteasome inhibition by MG132 indicating a primary effect on protein production and not protein stability (Figure S1D). These molecular and in vitro effects are reflected in the treatment of aggressive lymphomas in vivo. For example, CR-1-31 B treatment (0.25 mg/kg, 2 times per week for 2.5 weeks) results in growth impairment of suDHL-6 xenografts (*n* = 10, *p* < 0.05) (Figure 1E) and also in a primary, patient-derived MYC^+^/BCL2^+^ diffused large B cell lymphoma (DLBCL) xenograft (0.25 mg/kg, 3 times per week for ~4 weeks) (*n* = 5, *p* < 0.05) (Figure 1F,G). Consistent with our prior analysis of the in vivo toxicity [21], we find that the treatment was generally well tolerated and did not result in weight loss or mortality (Figure S1E). Hence, eIF4A1 inhibition could be a new and effective treatment for aggressive and MYC^+^/BCL2^+^ lymphomas.

### 2.2. NRF2 Activation Confers Resistance to EIF4A Inhibition

**Figure 2:**
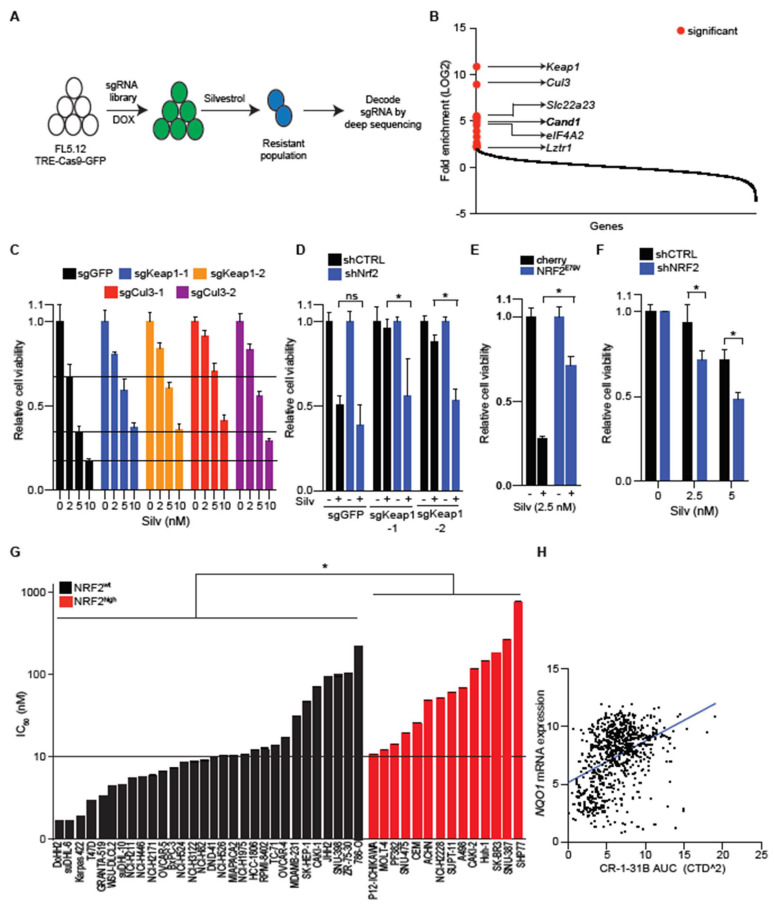
Genome-wide CRISPR screen identifies loss of NRF2 regulators as a cause of resistance to eIF4A inhibition. (**A**) Schematic of the genome-side CRIPSR screen designed to identify resistance mechanisms to eIF4A inhibitor; (**B**) change in guide RNA representation in silvestrol-treated cells (*n* = 3) relative to those treated with DMSO (*n* = 3); (**C**) viability of FL5-12 cells transduced with sgRNAs targeting GFP, *Keap1*, or *Cul3* and treated with either DMSO or silvestrol (48 h) as indicated; data are plotted as mean and s.d. from *n* ≥ 2; (**D**) *Nrf2* proficient and deficient FL5-12 cells with or without *Keap1* editing were treated with silvestrol as indicated, and cell viability was measured 48 h post treatment; error bars represent s.d. from *n* = 5; (**E**) cell viability of control and NRF2^E79V^ expressing suDHL-6 lymphoma cells treated with silvestrol as indicated; error bar is s.d. from 3 replicates; (**F**) *NRF2* proficient and deficient Ly19 cells were treated with silvestrol as indicated and cell viability was measured 48 h post treatment; data represent mean and s.d. from *n* = 3; (**G**) waterfall plot of silvestrol or CR-1-31 B IC_50_ values for indicated NRF2 wild-type (black) or mutant (red) cell lines; error bars represent s.d. from *n* ≥ 3; (**H**) correlation between CR-1–31 B sensitivity and expression of the NRF2 target gene *NQO1* derived using DepMap database (*n* = 770). ns = significant and * represents *p*-value < 0.05.

To identify genetic requirements for sensitivity to eIF4A inhibition, we performed an unbiased, pooled, genome-wide CRISPR/Cas9 screen in the context of silvestrol treatment (Figure 2A). Briefly, we engineered FL5-12 cells, a silvestrol-sensitive immortalized murine B cell line (IC_50_, ~5 nM), to express Cas9 in a doxycycline dependent manner (FL5-12-TRE-Cas9-GFP) and transduced them with a genome-wide CRISPR library (~90,000 sgRNAs targeting ~19,000 genes) [28]. We induced Cas9 expression for ~5 days to enable gene editing. We then treated the edited cells with IC_90_ concentration of silvestrol three times allowing for recovery between cycles and then sequence identified sgRNA vectors integrated in surviving cells. Comparison with mock (DMSO) treated controls identified a significant enrichment (*q* < 0.05) of sgRNAs targeting *Keap1* (5/5 sgRNAs), *Cul3* (4/5 sgRNAs), and the CUL3 recycling factor *Cand1* (3/5 sgRNAs), and three against another CUL3 interacting factor *Lztr1* [29] (Figure 2B). Additionally, we see three sgRNAs targeting the xenobiotic uptake and secretion factor Slc22 a23 indicating a role for it in silvestrol import [30]. We also detect enrichment of 3/5 sgRNAs targeting eIF4A2, an eIF4A1 paralog that is known to bind and repress translation of eIF4A1 targets, akin to chemical inhibitors such as silvestrol [31] (Figure 2B; the complete results of the screen are shown in Table S1). Therefore, our screen results indicate a new role for NRF2 regulators in silvestrol sensitivity.

The proteins encoded by *Keap1*, *Cul3*, and *Cand1* form the E3 ligase complex that targets NRF2 for proteasomal degradation [32]. The enriched sgRNAs effectively target *Keap1* and *Cul3*, respectively, and inactivate these genes (Figure S2A) [14]. This leads to NRF2 stabilization, target induction (NQO1, TXNRD1), and diminished silvestrol sensitivity (Figure 2C, Figures S2B–E and S3). In principle, *Keap1* may have NRF2-independent functions and we tested to what extent silvestrol resistance depends on NRF2 by silencing *Nrf2* in *Keap1* edited FL5-12 cells which readily restored silvestrol sensitivity to wild-type levels (Figure 2D and Figure S2F). Consistently, expression of the stabilized NRF2^E79V^ allele, but not a transcriptionally dead variant (NRF2^E79V−ΔNEH1^) confers silvestrol resistance (Figure S2G,H). Similarly, expression of NRF2^E79V^ in the MYC^+^/BCL2^+^ SuDHL-6 lymphoma cells protects them against silvestrol treatment (Figure 2E). NRF2-activating mutations are rare in untreated lymphomas; however, the human DLBCL OCI-Ly19 cell line carries an endogenous point mutation in *CUL3* gene (CUL3^D698G^) and is far less sensitive to silvestrol than other lymphoma lines and, again, this effect depends on NRF2 such that its knockdown augments silvestrol sensitivity (Figure 2F and Figure S2I). The protective effect of NRF2 activation is not limited to these select examples but found across multiple tumor types. For instance, 15 out of 30 cell lines that do not have NRF2-activating mutations show better silvestrol sensitivity than any of the 14 cell lines with known NRF2-activating mutations (Figure 2G). Similarly, expression of the canonical NRF2 target gene *NQO1* is inversely correlated (*n* = 770, *R* = 0.4, *p* < 0.00001) with CR-1–31 B sensitivity in the DepMap dataset (Figure 2H). Consistently, *KEAP1* editing in NRF2 wild-type HepG2 cells decreases and, conversely, *NRF2* loss in NRF2-stabilized KEAP1^D236H^ mutant H460 cells increases silvestrol sensitivity (Figure S2J,K). Hence, NRF2 activation attenuates the therapeutic effect of eIF4A inhibition in lymphomas and other cancers.

### 2.3. NRF2 Increases Translation of the eIF4A-Dependent mRNAs MYC and BCL2

**Figure 3:**
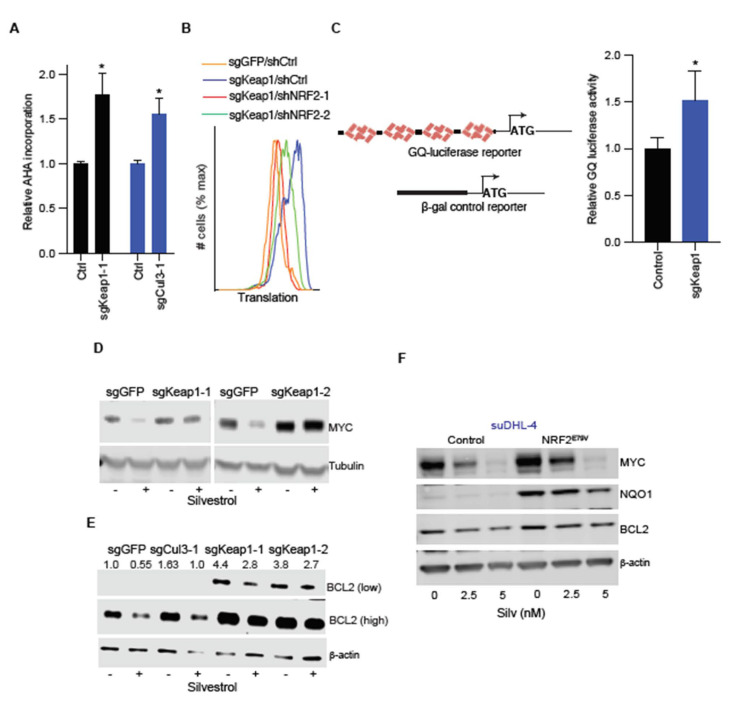
NRF2 broadly augments protein production and disproportionately increases eIF4A-dependent translation of MYC and BCL2. (**A**) AHA labeling to determine global translation in wild-type, *Keap1*, or *Cul3* edited FL5-12 cells, as indicated; data are mean and s.d. from *n* = 4; (**B**) AHA labeling to determine global translation in *Nrf2* proficient and deficient FL5-12 cells with or without *Keap1* editing, as indicated; (**C**) translational efficiency of GQ-luciferase reporter in unedited and *Keap1* edited FL5-12 cells; data are represented as the ratio of luciferase (encoded by transcript with 4 tandem GQ elements) to β-galactosidase (encoded by transcript without GQ elements) and plotted as means and s.d. from *n* = 3 replicates; (**D**) lysates from *Keap1* proficient and deficient FL5-12 cells treated with DMSO or silvestrol (10 nM, 24 h) were immunoblotted and probed with indicated antibodies; (**E**) lysates from wild type, *Cul3*, or *Keap1*-edited FL5-12 cells treated with DMSO or silvestrol (10 nM, 24 h) were immunoblotted and probed with indicated antibodies; (**F**) lysates from mCherry or NRF2^E79V^ expressing suDHL-4 cells treated with silvestrol (48 h) or not, as indicated, were immunoblotted and probed with indicated antibodies. Original immunoblot images are available in Figure S6. * represents *p*-value < 0.05.

An interesting, recent study showed that NRF2 augments baseline translation by countering oxidation of cysteine residues in several translational factors and ribosomal proteins [15]. Therefore, we examined the possibility that NRF2 may affect either global or eIF4A-dependent translation and counter the inhibitory effect of silvestrol. First, metabolic labeling with AHA showed a substantial increase in global protein synthesis in FL5-12 cells upon *Keap1* or *Cul3* editing (Figure 3A). This effect required NRF2 and knockdown with two independent short hairpin (sh) RNA’s reverted translation to basal levels in *Keap1*-edited cells (Figure 3B). Consistently, expression of the constitutively active NRF2^E79V^, but not the transcriptionally dead NRF2^E79V−ΔNEH1^ allele, increased translation in FL5-12 cells (Figure S3A). We next wondered whether *Keap1* edition could restore translation in silvestrol treated FL5-12 cells. As expected, silvestrol blocks protein synthesis in FL5-12 cells and *Keap1* deficiency largely mitigates this effect (Figure S3B).

Next, we examined to what extent NRF2 affects eIF4A-dependent translation compared to global translation. Briefly, we utilized an eIF4A1-dependent luciferase reporter comprising four tandem GQ elements and compared the effect on a control, eIF4A1-independent β-galactosidase reporter in *Keap1* proficient and deficient cells (Figure 3C). Compared to the global translation reporter, we see a 1.5-fold increase in eIF4A-dependent GQ-luciferase reporter activity in *Keap1* edited FL5-12 cells (*n* = 3, *p* < 0.05) (Figure 3B). Consistently, by comparing mRNA and protein levels, we observe increased baseline translation of eIF4A-dependent mRNAs with multiple GQ elements in their 5′ UTRS, such as MYC (six GQ elements) and BCL2 (five GQ elements) [21] (Figure 3D,E and Figure S3C–E). The effect is even more pronounced upon eIF4A inhibition by silvestrol (10 nM, 24 h), which causes rapid loss of MYC and reduction in BCL2 proteins, reflecting different protein half-lives (Figure 3D,E). On the other hand, MYC and BCL2 protein remained nearly unchanged in silvestrol treated with the *Keap1*-deficient cells (Figure 3D,E). Similarly, *Cul3* editing decreases the effect of silvestrol on BCL2 translation albeit to a lesser degree (Figure 3E). We observed the same effect by expressing a constitutively active NRF2^E79V^ allele in different human lymphoma cells (suDHL-4, suDHL-6, and suDHL-10) (Figure 3F and Figure S3F). Similarly, CUL3^D698G^ Ly19 cells that are resistant to eIF4A inhibition show ~30% reduction in MYC protein upon silvestrol treatment (5 nM, 24 h), and this effect depends on NRF2 (Figure S3G). We also examined other potential mechanisms of NRF2-mediated silvestrol resistance. For example, NRF2 has been implicated in radiation and chemotherapy resistance by neutralizing oxygen radicals. However, compared to a known ROS inducer (pycocynin), we see no increase in ROS with silvestrol (Figure S3H). Furthermore, it is known that silvestrol is a substrate of the exporter [33] (encoded by *Abcb1 a* and *Abcb1 b* genes); however, these exporters are not expressed in *Keap1* proficient or edited FL5-12 cells (not shown). Hence, NRF2 activation increases global protein production and disproportionately favors eIF4A-dependent mRNAs, including MYC and BCL2.

### 2.4. FN3K Inactivation Reverses NRF2 Driven Drug Resistance

**Figure 4:**
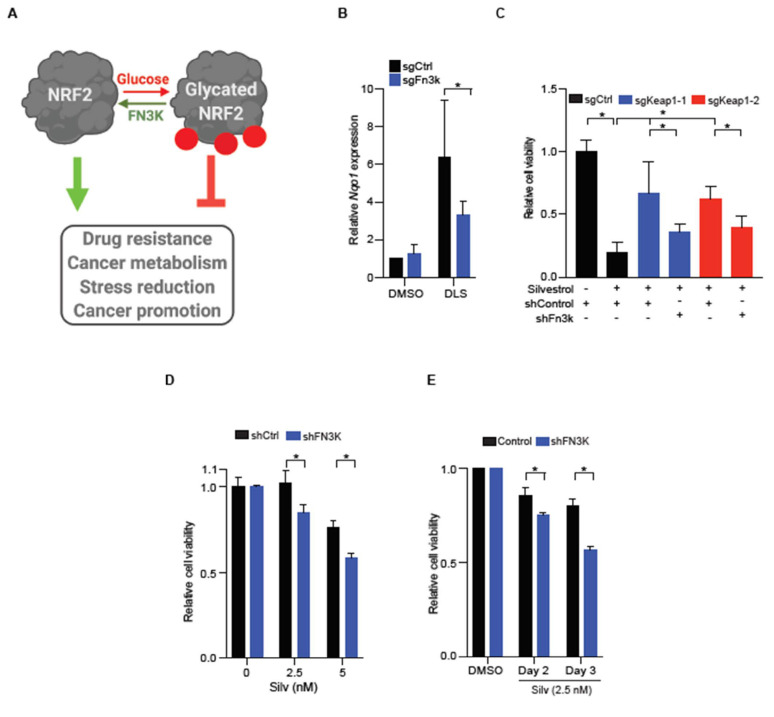
FN3K is required for NRF2-driven silvestrol resistance. (**A**) Schematic showing the regulatory role of glucose dependent glycation and FN3K catalyzed deglycation in NRF2 function; (**B**) expression of the NRF2 target gene *Nqo1* in FL5-12 cells transduced with control or *Fn3k* specific sgRNA/Cas9 treated with DMSO or NRF2 activator DLS, as indicated; error bars represent s.d. from *n* = 3; (**C**) cell viability of *Fn3k*-proficient and deficient FL5-12 cells, with or without *Keap1* editing treated with DMSO or silvestrol (48 h), as indicated; error bars represent s.d. from *n* = 5; (**D**) *FN3K*-proficient and deficient Ly19 cells were treated with DMSO or silvestrol (48 h), as indicated, and cell viability was measured; error bars are s.d. from 3 replicates; (**E**) *FN3K*-proficient and deficient Ly19 cells were treated with DMSO or silvestrol (2.5 nM) and cell viability was measured after 48 and 72 h post treatment; error bars are s.d. from *n* = 3. * represents *p*-value < 0.05.

We have recently shown that NRF2 and other proteins undergo a non-enzymatic post-translational modification called glycation, which refers to the covalent addition of simple sugars such as glucose onto proteins [14]. Glycation has a striking effect on NRF2 function, and the glycated NRF2 is unstable and functionally impaired; the de-glycating enzyme fructosamine-3-kinase (FN3K) phosphorylates and triggers removal of the attached sugars and this restores NRF2 function (Figure 4A) [14]. First, we confirmed that *Fn3k* loss suppressed NRF2 function in FL5-12 cells. We measured the expression of the canonical NRF2 target gene *Nqo1* following NRF2 activation with DL-sulforaphane (DLS). In *Fn3-k*-proficient cells, we see a ~6-fold increase in *Nqo1* after DLS treatment (2 μM, 24 h), which is reduced by ~50% upon *Fn3k* editing (Figure 4B and Figure S4A). Next, we tested how *Fn3k* status affects silvestrol sensitivity in *Keap1* proficient and deficient FL5-12 cells. Briefly, *Fn3-k*-proficient *Keap1*-edited FL5-12 cells are relatively protected from silvestrol (5 nM). However, concurrent silencing of *Fn3k* in the *Keap1*-edited cells largely reverses the NRF2-driven resistance phenotype (Figure 4C and Figure S4B). Similarly, CUL3^D698G^ mutant Ly19 cells depend on FN3K for NRF2 target gene NQO1 expression, and NRF2-mediated MYC and BCL2 maintenance and silvestrol resistance (Figure 4D,E, Figures S3G and S4C).

## 3. Discussion

Our findings provide new insight into the potential utility of eIF4A RNA helicase inhibitors in the treatment of human cancers and including aggressive lymphomas. Briefly, the eIF4A/DDX2 helicase is required to translate a subset of mRNAs with highly structured 5′ UTRs [21]. This is part of a conserved mechanism and controls the production of proteins such as MYC, BCL2, and other oncoproteins [21]. We and others have shown the feasibility of eIF4A inhibitors against a range of cancers including leukemia, non-Hodgkin’s lymphoma—especially MYC^+^/BCL2^+^ lymphomas, and perhaps more modestly against pancreatic cancer [21,34,35]. Recently, the eIF4A inhibitor eFT226 (Zotatifin) has entered clinical trials for advanced solid tumors, particularly those presenting with activation of receptor tyrosine kinases or with KRAS mutations [36]. We find that this type of therapy is highly effective against aggressive and MYC^+^/BCL2^+^ lymphoma cells in vitro and in vivo. On the other hand, we know that MDR1, the p-glycoprotein exporter, can expel silvestrol and its analogues and lead to resistance [33]. In addition, we identify three different genetic lesions that activate NRF2 and that attenuate the therapeutic effect of this class of therapeutics. NRF2-activating mutations are rare in untreated lymphomas; however, they have been associated with chemo- and radiation resistance in lymphoma [37,38], leukemia [39], and in solid tumors [40]. NRF2-mediated drug resistance is thought to result from its ability to counter treatment-related ROS production. However, silvestrol and its analogues do not directly produce ROS suggesting a distinct mechanism. Indeed, NRF2 is known to augment global protein production by reducing key translation factors such as eEF2 [15]. Moreover, we find that NRF2 specifically increases the production of eIF4A-dependent oncoproteins such as MYC and BCL2. This directly counters a critical part of the therapeutic mechanism of this class of drugs and further highlights the importance of blocking NRF2 action in cancer. In this regard, we recently showed that the activity and stability of NRF2 depend on the continuous removal of sugar adducts (de-glycation) that is triggered by the kinase FN3K [14]. Consistently, we see that inhibition of FN3K can neutralize the drug resistance effects of NRF2 activation. Going forward, FN3K inhibition emerges as a new strategy to attenuate NRF2 functions in cancer that include broad resistance to different treatment modalities.

## 4. Materials and Methods

### 4.1. Cell Culture and Animal Modeling

FL5-12 [14], suDHL-4, suDHL-6, suDHL-10, Ly8, and Ly19 cells (American Type Culture Collection (ATCC), Manassas, VA, USA) were maintained in RPMI-1640 (Rosewell Park Memorial Institute-1640) (MSKCC core facility, New York, NY, USA) supplemented with heat inactivated fetal bovine serum (10%) (Sigma-Aldrich F8317, St. Louis, MO, USA), L-glutamine (2 mM) (Gibco, Montgomery County, MD, USA), penicillin/streptomycin (Gibco), and plasmocin (Invivogen, San Diego, CA, USA). In addition, FL5-12 media contained IL3 (1 ng/mL) and WEHI (ATCC) conditioned media. HepG2, H460, and 293T (ATCC) were cultured in DMEM (Dulbecco’s Modified Eagle Medium) (MSKCC core facility, New York, NY, USA) supplemented with heat inactivated fetal bovine serum (10%), L-glutamine (2 mM), penicillin/streptomycin, and plasmocin. For lentivirus preparation, 293T cells were transfected with psPAX2, pVSV.G, and target construct (2:1:2). Target cells were transduced with concentrated (Lenti-X concentrator, Clonetech, Mountainview, CA, USA) or dilute virus as needed in the presence of polybrene (4–8 μg/mL).

Approximately 5 million suDHL-6 and DLBCL PDX were engrafted in nude and NOD/SCID/IL2 Rγ^−/−^ (NSG) mice, respectively, in 50% matrigel. Tumor measurements were done periodically, and volumes were calculated using the Equation (1):

1/6 × π × length × width^2^/1000 (1)

where π is 3.14.

Animals were randomized prior to treatments and tumor measurements were performed in a blinded manner, i.e., person injecting/measuring tumors did not know the experimental conditions. All mice used in this study were ~8-week-old females bought from Jackson laboratories (Farmington, CT, USA).

### 4.2. IC_50_ Determination

The large-scale sensitivity screen in 183 cell lines shown in Figure 1B was conducted by Horizon Discovery (Waterbeach, UK). For in-house IC_50_ determinations, cells were treated in 96-well plates with silvestrol or CR-1-31 B at concentrations ranging from 1 to 5 μM and cell viability was assessed by quantifying ATP content at 24, 48, and 72 h post treatment using CellTiter-Glo^®^ (Promega, Madison, WI, USA). IC_50_ values were determined using Prism 7. All other cell viability assays were also carried out using CellTiter-Glo^®^ assay (Promega).

### 4.3. CRISPR Screen and Analysis

FL5-12 cells were used for a genome-wide CRISPR screen to identify genetic determinants of silvestrol. Briefly, we first generated an FL5-12 cell line that expresses Cas9 in a doxycycline-dependent manner. Following infection with a genome-wide CRISPR library in triplicate, we performed three sequential cycles of treatment with silvestrol (10 nM) or DMSO (Dimethyl Sulfoxide) allowing brief period of recovery. Silvestrol-treated cells were washed to eliminate dead cells and DMSO treated cells were split 1:10 during each recovery period. After the third cycle, we harvested genomic DNA from silvestrol and DMSO treated samples, and integrated sgRNA sequences were amplified and deep sequenced as previously performed [14]. The sgRNAs enriched in silvestrol-treated cells relative to DMSO were identified as before [14]. Briefly, all reads were trimmed using fastx_trimmer from fastx_toolkit (version 0.0.13) to extract the region of interest, then collapsed with fastx_collapser to obtain a table of raw counts. This table of counts was then joined with the sgRNA library sequences to assign the read counts to the corresponding sgRNA. Differential Analysis on the resulting raw read counts table was performed using the R package (version 3.4.0) DESeq, with nbinomTest and local fit [14].

### 4.4. Gene or Protein Expression and Reporter Assays

Quantitative RT-PCR (qPCR) and immunoblots were performed as before [14]. We used the following taqman probes for qPCR analysis: HUMAN—*NRF2* (Hs00975961_g1, Life Technologies, Carlsbad, CA, USA), *FN3K* (Life Technologies, Hs_00223368_m1), *GUSB* (Life Technologies, 4333767 F), and *ACTB* (Life Technologies, 4332645); MOUSE—*Nqo1* (Life Technologies, Mm01253561_m1), *Nrf2* (Life Technologies, Mm00477784_m1), *Fn3k* (Life Technologies, Mm00445584_m1), *cMyc* (Life Technologies, Mm00487804_m1) and *Actb* (Life Technologies, Mm00607939_s1). For immunoblotting, we used the following antibodies from Cell Signaling (Danvers, MA, USA): NQO1 (CST, 62262), MYC (CST, 5605), BCL2 (CST, 3498), KEAP1, (CST, 8047), CUL3 (CST, 2759), GAPDH (CST, 5174), and β-actin (Sigma-Aldrich A5441 St. Louis, MO, USA). Proteins were visualized using LI-COR Lincoln, NE, USA detection system after incubation with following secondary antibodies: Goat anti-rabbit-IR800 (LI-COR, 926–32211) and Goat anti-mouse-IR680. We used the following reporter assays as per manufacturer’s instructions: Luciferase-Glo (Promega) and Beta-glo (Promega). T7 endonuclease assays were conducted to confirm gene editing (NEB, Ipswich, MA, USA).

### 4.5. Flow Cytometry and AHA Labeling

For flow cytometry, FL5-12 cells were fixed, permeabilized (00–5521-00 and 00–8333, eBioscience^TM^, San Diego, CA, USA) and stained with PE-conjugated NRF2 antibody according to manufacturer’s protocol (CST, Danvers, MA, USA). AHA Click-IT^®^ labeling was performed according to manufacturer’s recommendations (C10102, Thermo Fisher, Waltham, MA, USA). Briefly, cells were incubated in methionine free media for 1 h under tissue culture conditions. Subsequently, AHA was added at a final concentration of 25 μM, and cells were allowed to incorporate it in nascent proteins for 1 h under tissue culture conditions. Cells were then washed, fixed, and permeabilized using manufacturer’s protocol (eBioscience, 00–5521-00). Cells were then incubated in fluorescent alkyne to label the AHA-incorporated proteins and analyzed using Guava bench-top flow cytometer.

### 4.6. Immunohistochemistry

Immunohistochemistry (IHC) was performed as previously described [32]. Briefly, tissue microarray (TMA) encompassing 1.5 mm formalin-fixed, paraffin-embedded (FFPE) tissue specimens from 34 patients diagnosed with DLBCL were used to obtain 4 μm sections for subsequent IHC using the Ventana BenchMark XT platform (Ventana, AZ, USA). We used the following antibodies as per the manufacturer’s recommendation; MYC (Y69, Epitomics, Burlingame, CA, USA), BCL2 (Clone 124, Dako, Santa Clara, CA, USA), and p-p70S6K (CST, 2708). Slides were evaluated by pathologists at the Memorial Sloan Kettering Cancer Center and scored based on staining intensity (0–1 = negative, 2–3 = positive).

### 4.7. Bioinformatics and Statistical Analysis

DepMap analysis was carried out using the online portal (https://depmap.org/portal/). All data presented here are from *n* ≥ 2 biological replicates and error bars represent either standard deviation unless otherwise mentioned. A Student *t* test with Welch correction was conducted using Prism (v.8.0). Statistical significance for screen was in-built in DESeq2 algorithm.

## 5. Conclusions

Our findings have important implications for treatment of human cancers with inhibitors of the eIF4A RNA helicase and for the role of NRF2 as a broad-acting drug resistance mechanism. Importantly, eIF4A inhibitors are entering clinical trials and it is important to identify and understand predictors of sensitivity. While NRF2 activating lesions are rare in lymphoma, they are very frequent in advanced solid tumors. Therefore, NRF2 status may help stratify patients who are more likely to respond favorably to eIF4A targeted therapies. Finally, future combination therapies that include FN3K inhibitors may be useful in reverting the NRF2-driven multidrug resistance programs.

## Data Availability

No new data were created or analyzed in this study. Data sharing is not applicable to this article.

## Supplementary Materials

The following are available online at https://www.mdpi.com/2072-6694/13/4/639/s1, Figure S1: Lymphomas are extremely sensitive to eIF4A inhibition, Figure S2: NRF2 activation antagonizes drug response to silvestrol, Figure S3: NRF2 promotes translation of eIF4A targets MYC and BCL2, Figure S4: FN3K knockdown reverts silvestrol sensitivity in NRF2 mutant cells, Figure S5: Original Western blot image of Figure 1D. Figure S6: Original Western blot images for Figure 3D–F, Table S1: Complete results of the screen.
